# Morphometric Evaluation of Interrenal Gland and Kidney Macrophages Aggregates in Normal Healthy Rainbow Trout (*Oncorhynchus mykiss*) and after Bacterial Challenge with *Yersinia ruckeri*

**DOI:** 10.1155/2014/210625

**Published:** 2014-02-24

**Authors:** Michela Gregori, Vincenzo Miragliotta, Roberto Leotta, Stefano Cecchini, Marino Prearo, Francesca Abramo

**Affiliations:** ^1^Department of Veterinary Sciences, University of Pisa, Viale delle Piagge 2 56124, Pisa, Italy; ^2^Department of Animal Production Sciences, University of Basilicata, 85100 Potenza, Italy; ^3^Experimental Zooprophylactic Institute of Piemonte, Liguria and Valle D'Aosta, 10154 Turin, Italy

## Abstract

Macrophage aggregates (MA) occur in various organs of fish as discrete aggregations of pigmented macrophages. The study presented herein investigates the quantitative modifications from normal anatomical condition, of interrenal gland (IG) and kidney MA in six treatment groups of adult rainbow trout submitted to either specific or aspecific immune stimulation and subsequently challenged with *Yersinia ruckeri*. Routinely stained tissue sections from both IG and kidney were analysed. The percentage of tissues occupied by MA and the MA density (number/mm^2^) were calculated on at least 10 randomly selected nonoverlapping fields taken from each tissue section. MA morphometric findings from challenged fish were compared to those from a control group. Results showed that fish from control group displayed a statistically significant (*P* < 0.05) higher percentage of tissue occupied by MA and MA density. Among different treatment groups, anti-*Yersinia ruckeri* immunized fish, which did not show clinical signs of disease after bacterial challenge, displayed higher values of morphometric parameters compared with symptomatic fish from other groups. Our study demonstrates that the quantification of the area occupied by MA might be an efficient parameter to evaluate the general condition of a salmonid population since it positively correlates with the health status and negatively with stress factor such as the acute bacterial infection.

## 1. Introduction

Macrophage aggregates (MA) are discrete aggregations of pigmented macrophages occurring primarily in hematopoietic and hepatic tissues of teleosts fish [1, 2]. There are several reports on the MA in a wide range of fish [3–6]. In Clupeiformes and Salmoniformes, MA are difficult to define because they are small, poorly organized, and irregularly shaped [1]. These structures are easily visualized in histological sections because of the presence of pigments, such as hemosiderin, melanin, and ceroid/lipofuscin, which range in colour from gold to brown to black in H&E stained slides [1, 7]. The morphological appearance of the MA may vary in different physiological and pathological conditions within the same species, such as age [7], starvation and tissue breakdown [8], iron and haemoglobin metabolism [9], pathological and inflammatory conditions [1], and immunological processes, including antigen trapping [1, 9]. MA have been suggested as a possible biomarker of the health status of wild fish populations [1, 10]. In particular, in some ecotoxicology studies, fish have been used as sentinel species and MA as biomarker for nonspecific contaminant exposure [7, 11, 12]. This broad application of MA investigation for assessment of fish and environmental health has been well-documented in many species and it has been recently investigated also for salmonids [2].

On the other side the utility of MA morphological modifications as a histopathological bioindicator or biomarker has been criticized by some researchers as being too nonspecific; others consider that too many variables are involved in alteration of MA parameters to be of value [7]. Moreover, studies on the relationship between status of MA and infectious diseases have reported conflicting results. Some authors indeed reported an increase of MA in association with the cellular response to a variety of infections [13, 14], while others recorded the opposite [15]. Agius and Roberts [1] also suggested that increases in pigment content are also suggestive of catabolic, toxic, or otherwise stressful events.

In the study presented herein we investigated the morphometric modifications occurring in IG and kidney MA in six groups of adult rainbow trout *(Oncorhynchus mykiss)* that have been submitted to a stimulation of the immune system and subsequently challenged with *Yersinia ruckeri* (*Yr*). *Yr* was selected for its known ability to cause enteric redmouth disease, which can represent a problem in salmonid aquaculture [16]. We compared morphometrical findings from *Yr*-challenged fish with data obtained from a control group. In this study we assumed that kidney would have been a suitable organ to perform the MA morphometric analyses. We chose to sample both anterior and posterior kidney for the different physiological roles they play: anterior kidney is indeed a homologous of mammalian adrenal cortex, that is, Interrenal Gland (IG)—and also harbours limphohaematopoietic tissue, chromaffin cells, and MA; the posterior portion is mainly devoted to maintaining the hydroelectrolytic balance [17].

The aim of the study was twofold: (1) to evaluate the MA anatomical distribution within IG and kidney of healthy rainbow trout and (2) to determine whether any eventual difference in MA morphometric findings may correlate with a preliminary specific and aspecific stimulation of the immune system and with the health status in different groups of *Yr*-infected fish.

## 2. Materials and Methods

### 2.1. Animals and Experimental Design

A batch of two hundred fifty female rainbow trout (weight 370 ± 50 g; age 12 months; size 17,5 ± 1 cm) was used in the study. Two hundred ten fishes were size-selected from the initial batch and randomly assigned to six treatment groups (A–F) and one control group of 30 animals each, as described below. During the experimental procedure trout were kept in circular tanks (*Ø* 2 meters), at a constant temperature of 14.5°C, with a water flow of 1.5 litres/second and a constant dissolved oxygen concentration of 7.5 ppm; fishes were fed with extruded commercial diet (1% body-weight) throughout the study duration.

### 2.2. Experimental Groups

Fish from different groups were treated as follows:

 Group A: intraperitoneally (ip) injected with 500 *μ*g of human gamma globulin (HGG, Sigma, Milan, Italy) dissolved in 0.1 mL of sterile 0.1 M phosphate buffered saline (PBS) and emulsified in an equal volume of Freund's Complete Adjuvant (FCA);
 Group B: intraperitoneally injected with 0.1 mL of sterile PBS emulsified with 0.1 mL of FCA;
 Group C: intraperitoneally injected with 500 *μ*g of HGG dissolved in 0.1 mL of PBS;
 Group D: intraperitoneally injected with 0.2 mL PBS;
 Group E: intraperitoneally injected with 0.1 mL of anti-*Yr* vaccine (Aquavac bocca rossa, Schering Plough);
 Group F: intraperitoneally injected with 0.1 mL of anti-*Yr* vaccine emulsified in 0.1 mL of FCA;
 Ctrl: control group animals were untreated.

### 2.3. Bacterial Challenge with *Yersinia ruckeri*

A virulent strain of *Yersinia ruckeri* (ATCC, *Yr* Serovar I) was used in the experimental infection. Challenge was performed by* ip *injection. Inocula were prepared as follows: colonies of *Yr* were diluted in 0.85% physiological saline solution to the appropriate concentration corresponding approximately to 1.5 × 10^7^ ufc/mL (DL_50_). Challenge was performed 40 days after the primary treatment reported above (groups A–F), by injection of 0.1 mL of bacterial suspension of *Yr *(containing 1.5 × 10^6^ ufc/mL for fish). Fish from Ctrl group were not inoculated with the pathogen. Feeding did not change among treatments.

### 2.4. Clinical Evaluation

Fish from different treatment groups were clinically monitored daily up to 14 days after bacterial challenge.

### 2.5. Fish Collection

Five fish randomly selected from each group were sacrificed by immersion in a solution of tricaine methane sulfonate (MS-222, Sigma Aldrich) (>100 mg/liter) and submitted to tissue sampling of IG and posterior kidney. The remaining 25 fishes were used for analyses unrelated to the present study.

### 2.6. Tissue Processing

Samples were collected from anterior (IG) and posterior kidney, fixed in 10% buffered formalin, and processed for histological examination as reported [18–21]. Sections of 5 *μ*m thickness were thus Haematoxylin & Eosin (H&E) stained for morphometric analysis.

### 2.7. MA Morphometric Analysis

**Figure 1:**
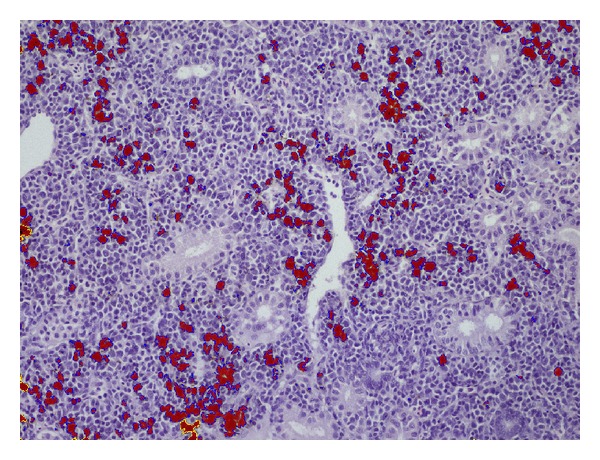
Rainbow trout (*Oncorhynchus mykiss*). Posterior kidney section in a trout from group F after software selected the pigmented area of interest.

Quantitative MA analysis was performed on at least 10 randomly selected, nonoverlapping fields taken from both IG and posterior kidney sections at 10x magnification with a light microscope (Nikon Eclipse 80i, Nikon, Tokyo, Japan). A computerized image analysis software (Lucia software, Nikon, Tokyo, Japan) was used for quantitative evaluation of MA. The percent area occupied by MA and the MA number per mm^2^ (MA density) were calculated from each captured field. To perform quantification, representative MA were selected on the basis of their colour as shown in Figure 1. Briefly, on a captured image a threshold was selected for colour (by clicking on MAs) and for size (manual tuning). The threshold performance was visually evaluated on at least 10 pictures. The best performing thresholds (colour and size) were included in an automated macroinstruction that gave number of objects, measured area (total area of the picture), and MA's area as output. MA density (number of objects/mm^2^) and area occupied by MA were thus calculated. Since aggregates are hardly definable in salmonids, we assumed that an MA must include more than 2 pigmented macrophages. For this reason, individual scattered macrophages were manually excluded from the final area occupied by MA.

### 2.8. Statistical Analyses

Statistical analysis (ANOVA) was conducted fitting a hierarchical model using an SLS (Standard Least Square) estimation algorithm (JMP, 2002—The Statistical Discovery Software, SAS Institute Inc.). Data was analysed on ln⁡(log⁡ natural) transformed data with the following model:

(1) $$\begin{array}{c} y_{iklm}=\mu +S_{i}+B_{k}+F{\left(B\right)}_{kl}+\epsilon_{iklm}, \end{array}$$

where *y*_*iklm*_ was the number of objects (or the area fraction), *m*th observation of the *l*th individual, of the *k*th group of treatment, and of the *i*th part of kidney; *μ* was the general mean; *S*_*i*_ was the fixed effect of the *i*th site, that is, IG and posterior kidney—(*i* = 2); *B*_*k*_ was the fixed effect of the *k*th group of treatment (*k* = 7); *F*(*B*)_*kl*_ was the fixed effect of the *l*th individual, nested in the *k*th group of treatment; *ϵ*_*iklm*_ represents the residual error.

Mean contrast was conducted using Tukey HSD test for multiple comparisons. Data were analyzed for a reduced model without the *S*_*i*_ (site) since IG and posterior kidneys are morphophysiologically different organs. Distribution of residuals was tested for normality with the Shapiro-Wilk test. Probability (*P*) values less than 0.05 were considered significant.

## 3. Results

### 3.1. Clinical Investigation

Fish from groups A, B, C, and D displayed clinical signs of disease (symptomatic groups) including lethargy (ranging from mild to severe), skin darkening, abdominal distension, severe enteritis, and exophthalmia. Signs of disease were not evident until day 2-3 after challenge. Fish from groups E, F (vaccinated), and Ctrl group appeared clinically normal (no external lesions; behaviour and feeding were normal; no mortality or morbidity was observed).

### 3.2. MA Morphometric Analysis

MA number/mm^2^ and MA area fraction were significantly different among tested groups (*F*_6,1122_ = 89,3389*P* < 0.0001 and *F*_6,1122_ = 207,2201*P* < 0.0001, resp.). More precisely, both parameters resulted to be decreased in *Yr* infected fish. Detailed results of the Tukey HSD test are reported in Table 1.

*Interrenal Gland MA Area Fraction (%).* Fish from Ctrl group displayed a statistically significant higher percentage of tissue occupied by MA when compared with all the other groups (*P* < 0.05). Fish from groups E and F, which did not show clinical signs of disease (asymptomatic fish), displayed a higher percentage of pigmented area in the IG when compared with groups A, B, C, and D.

*Interrenal Gland MA Density (Number/mm* ^*2*^ *).* Ctrl group displayed the greatest mean values followed by groups E > C > F > D > B > A. A statistically significant difference was found between Ctrl group and all the other groups except for group E.

*Posterior Kidney MA Area Fraction (%). *In the posterior kidney again fish from Ctrl group displayed the greatest percentage of tissue occupied by MA compared with all the other groups and the differences were statistically significant (*P* < 0.05). Fish from groups E and F (asymptomatic fish) displayed a higher percentage of tissue occupied by MA than groups A, B, C, and D (symptomatic fish).

*Posterior Kidney MA Density (Number/mm* ^*2*^ *).* The greatest mean value was observed in the ctrl group (the differences with all the other groups were statistically significant), followed by groups: F > E > D > C > B > A.

Finally, in the Ctrl group both the percentage of tissue occupied by MA and the MA number/mm^2^ were higher in the posterior kidney than in the IG, and the differences were highly significant (*P* < 0.001).

**Figure 2:**
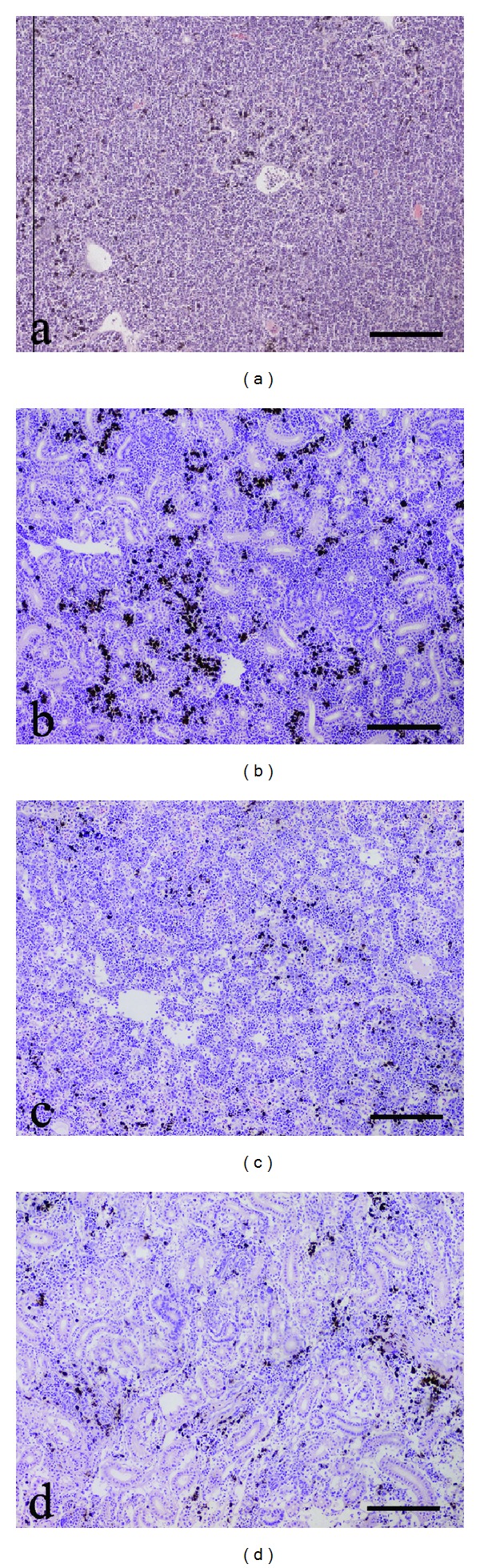
Rainbow trout (*Oncorhynchus mykiss*). Anterior (a) and posterior (b) kidney section, showing an extensive accumulation of macrophage aggregates (MA) in a normal trout from Ctrl group. Anterior (c) and posterior (d) kidney section, showing very few macrophages aggregates in a diseased trout from group C (H&E, scale bar = 200 *μ*m).

Figure 2 shows typical sections obtained from Ctrl group (Figures 2(a) and 2(b)) and symptomatic fish (Figures 2(c) and 2(d)).

## 4. Discussion

The main result of the present study is the evidence that *Yr* infection reduced both the number and the area occupied by MA in rainbow trout IG and posterior kidney leading to the conclusion that both organs are suitable for morphometric comparisons. Despite evaluation of MA in salmonids having been largely ignored due, presumably, to difficulties in quantifying the poorly organized structures present in these species of fish [1], the present study shows statistically reliable data that may be used in monitoring the general status of health of a salmonid population. Manual counting of MA is potentially ambiguous in salmonids that have poorly defined MA and is obviously time consuming. In this study we addressed this problem using the area occupied by MA pigments, ranging in colour from dark brown to black, rather than direct manual count of MA, and used an image analysis software to quantify the pigmented area. The method employed to automate pigment selection was found to be easy, quick, reliable, and as efficient as hand selection. Other investigators have used area occupied by MA pigments rather than number of MA aggregates to assess the health status of wild fish populations [2]; however, only a few of them [7] has added the number of MA per square millimeter as an additional parameter. In this study, we quantified both the area occupied by MA and their number/mm^2^.

The MA function has been associated with immunity (inflammatory and humoral responses) [15]; cell and compound storage, destruction and detoxification [22]; and iron recycling [7]. Moreover, pathogens induce MA morphological modifications, particularly parasites associated with chronic, focal infections [23]. As suggested in some papers [7, 15], it is important to first characterize the “normal” baseline of MA morphometry for the specific fish population of interest using fish from a control group. After this baseline has been established it can be used to determine variations that may correlate to environmental factors or fish health [15].

To the authors' knowledge there is evidence of a recent study performing a morphometric evaluation of macrophage aggregates in the kidney of rainbow trout based on the selection and the quantification of the pigmented area which was expressed as of tissue % occupied by MA [2]. In our study we observed lower values of MA pigmented area in the kidney of healthy rainbow trout from the control group compared to the results presented by the abovementioned paper. It is likely that the differences with our findings depend on factors such as age and fish size; consequently, our results are not “directly” comparable with those from Schwindt and colleagues. These latter authors also reported that the pigmented area was greatest in the kidney interstitium, with the spleen displaying about half that of the kidney and the liver showing 20 times less pigmentation than the kidney and 10 times less than the spleen. Based on these mentioned results we decided to evaluate IG (as a new organ) and kidney MA in our study [2].

Moreover, our investigation indicates that there was a significant difference in MA morphometry between IG and posterior kidney, in particular we observed greater values of MA pigmented area and density in the posterior kidney rather than in the IG. This finding adds value to our work since data were obtained from morphophysiologically different tissues that can thus be considered independent; as for the response to *Yr* infection: their analogous response increases reliability of observations.

We observed in the Ctrl group the greatest percentage of tissue occupied by MA and the highest number of MA. Results from statistical analysis demonstrated that the differences with all the other treatment groups of fish were statistically significant (*P* < 0.05). Moreover, we observed that both the percentage of tissue occupied by MA and the MA number were higher in groups E and F (clinically healthy fish) than in the other groups (A, B, C, and D) of diseased fish.

These results support the theory that MA dynamics in healthy and sick fish may be different and might be influenced by many factors [15]. Variations in the percentage of tissue area occupied by MA indicate stress on the physiological homeostatic mechanisms of the fish, and therefore an alteration of the health status of the fish [7].

The reduction in MA observed in *Yr-*infected diseased fish could be associated with a decreased phagocytic activity, with consequent decrease in MA.

Despite conclusions are limited to the animals used, due to the unreplicated nature of the study, our results demonstrated that: (a) quantitative MA pigments evaluation is applicable for salmonids, and potentially, for other fish species that do not display easily discernible MA; (b) both IG and kidney are suitable tissues for MA analysis; (c) variations in the MA morphometry may be used to monitor fish health. Other organs (i.e., liver and spleen) might be used to test the hypothesis that *Yr* infection leads to MA area and number decrease.

## Figures and Tables

**Table 1:** *Oncorhynchus mykiss.* Percentage (mean values) of tissue occupied by macrophages aggregates (MA) and MA number/mm^2^ in the interrenal gland and posterior kidney from six groups of *Yersinia ruckeri* infected rainbow trout (A, B, C D, E, and F) and a control group (Ctrl).

			Interrenal gland										Posterior kidney														
Groups	MA Area fraction (%)		Levels				Number of MA/mm^2^		Levels				MA Area fraction (%)		Levels					Number of MA/mm^2^		Levels					
	Mean	Std Error					Mean	Std Error					Mean	Std Error						Mean	Std Error						
A	0.79	0.4542			c		420.19	9.757				d	1.10	0.1432				d		440.86	10.335						f
B	0.84	0.4646			c		485.08	9.988			c	d	1.15	0.1482				d		518.44	10.697					e	
C	0.98	0.4625			c		708.50	9.940		b			1.41	0.1521			c			749.83	10.976				d		
D	1.51	0.5257		b			520.19	11.298			c		3.54	0.1691		b				856.22	12.208			c	d		
E	2.35	0.4653	a				777.72	9.994	a				3.44	0.1547		b				944.56	11.167		b	c			
F	1.76	0.5236		b			560.03	11.250		b	c		4.80	0.1581	a					1154.88	11.413		b				
Ctrl	2.49	0.6056				d	882.41	13.018	a				6.47	0.1901					e	1551.55	13.721	a					

Letters represent significant differences (*P* < 0.05) among groups of *Yersinia ruckeri*-infected fish (A, B, C, D, E, and F) and a control group (Ctrl) based on Tukey analysis run separately for the interrenal gland and the posterior kidney data. Levels not connected by the same letter are significantly different.
